# TRPV1-mediated UCP2 upregulation ameliorates hyperglycemia-induced endothelial dysfunction

**DOI:** 10.1186/1475-2840-12-69

**Published:** 2013-04-22

**Authors:** Jing Sun, Yunfei Pu, Peijian Wang, Sijiao Chen, Yu Zhao, Chan Liu, Qianhui Shang, Zhiming Zhu, Daoyan Liu

**Affiliations:** 1 Center for Hypertension and Metabolic Diseases, Department of Hypertension and Endocrinology, Daping Hospital, Third Military Medical University, Chongqing Institute of Hypertension, Chongqing, 400042, China; 2 Teaching and Research Office for Geriatric Disease, First Affiliated Hospital of China Medical University, Shenyang, 110001, China; 3 Institute of Clinical Medicine of Zunyi Medical College,Department of Cardiology, Affiliated Hospital of Zunyi Medical College, Zunyi, Guizhou, 563003, China

**Keywords:** TRPV1, Diabetes, Capsaicin, Endothelium, Oxidative stress, UCP2

## Abstract

**Background:**

Diabetic cardiovascular complications are characterised by oxidative stress-induced endothelial dysfunction. Uncoupling protein 2 (UCP2) is a regulator of mitochondrial reactive oxygen species (ROS) generation and can antagonise oxidative stress, but approaches that enhance the activity of UCP2 to inhibit ROS are scarce. Our previous studies show that activation of transient receptor potential vanilloid 1 (TRPV1) by capsaicin can prevent cardiometabolic disorders. In this study, we conducted experiments in vitro and in vivo to investigate the effect of capsaicin treatment on endothelial UCP2 and oxidative stress. We hypothesised that TRPV1 activation by capsaicin attenuates hyperglycemia-induced endothelial dysfunction through a UCP2-mediated antioxidant effect.

**Methods:**

TRPV1^-/-^, UCP2 ^-/-^ and db/db mice, as well as matched wild type (WT) control mice, were included in this study. Some mice were subjected to dietary capsaicin for 14 weeks. Arteries isolated from mice and endothelial cells were cultured. Endothelial function was examined, and immunohistological and molecular analyses were performed.

**Results:**

Under high-glucose conditions, TRPV1 expression and protein kinase A (PKA) phosphorylation were found to be decreased in the cultured endothelial cells, and the effects of high-glucose on these molecules were reversed by the administration of capsaicin. Furthermore, high-glucose exposure increased ROS production and reduced nitric oxide (NO) levels both in endothelial cells and in arteries that were evaluated respectively by dihydroethidium (DHE) and DAF-2 DA fluorescence. Capsaicin administration decreased the production of ROS, restored high-glucose-induced endothelial dysfunction through the activation of TRPV1 and acted in a UCP2-dependent manner in vivo. Administration of dietary capsaicin for 14 weeks increased the levels of PKA phosphorylation and UCP2 expression, ameliorated the vascular oxidative stress and increased NO levels observed in diabetic mice. Prolonged dietary administration of capsaicin promoted endothelium-dependent relaxation in diabetic mice. However, the beneficial effect of capsaicin on vasorelaxation was absent in the aortas of UCP2 ^-/-^ mice exposed to high-glucose levels.

**Conclusion:**

TRPV1 activation by capsaicin might protect against hyperglycemia-induced endothelial dysfunction through a mechanism involving the PKA/UCP2 pathway.

## Background

Reactive oxygen species (ROS) generated by hyperglycemia contribute to the development and progression of diabetic vascular complications and are linked to endothelial dysfunction [1-4]. Vascular nicotinamide adenine dinucleotide phosphate (NAD(P)H) oxidase activity, mitochondrial disturbance and the impairment of endothelial nitric oxide synthase (eNOS) activity all lead to oxidative stress in diabetes [3,5].

Given the importance of oxidative stress in diabetic vascular complications, anti-oxidative stress treatment is a very important intervention that has the potential to ameliorate hyperglycemia-induced vascular lesions. Up to now, several drugs, including statins and angiotensin II receptor blockers, have been reported to offer protection against oxidative stress in diabetic patients with vascular complications [2,4,6,7]. Non-pharmaceutical approaches, such as regular exercise, also have a beneficial effect in diabetic patients with vascular complications [8], but the underlying mechanisms of this effect are poorly understood.

Uncoupling protein 2 (UCP2) is a member of the mitochondrial anion carrier family that is widely expressed in a variety of tissues [9]. UCP2 is thought to function as a physiological regulator of mitochondrial ROS generation and may contribute to the prevention of diabetes [10,11]. A recent study conducted by our research group showed that UCP2 plays an important role in preventing salt-sensitive hypertension, which is associated with the suppression of superoxide production and the reservation of nitric oxide (NO) bioavailability in blood vessels [12]. Several studies have shown that administration of capsaicin or its analogue can increase UCP2 expression in adipose and hepatic tissues [13,14]. Multiple cyclic adenosine monophosphate (cAMP) response elements have been identified in the promoter region of human UCP2, and UCP2 expression is stimulated by the cAMP/PKA signal cascade [15]. Adiponectin has been shown to suppress high-glucose-induced ROS production in cultured human umbilical vein endothelial cells through a cAMP/PKA-dependent pathway [16]. Transient receptor potential vanilloid 1 (TRPV1), a polymodal nonselective cation channel, is expressed in sensory neurons and also present in nonneuronal tissues including blood vessels, is a highly selective stimulated by capsaicin [17]. Our previous studies suggest that activation of TRPV1 is a potential therapeutic target for obesity, hypertension, atherosclerosis and diabetes [18-21]. We also showed that prolonged dietary administration of capsaicin increased NO production remarkably and improved endothelial function through specific targeting of TRPV1-mediated PKA/eNOS phosphorylation [20]. Although it is well documented that oxidative stress contributes to the vascular complications in diabetes, the factors that play a role in protecting against the associated endothelial dysfunction remain to be discovered.

The present study was undertaken to test the hypothesis that TRPV1 activation by capsaicin attenuates hyperglycemia-induced endothelial dysfunction through a UCP2-mediated antioxidant effect. We also elucidated the underlying mechanism by which TRPV1 activation reduces oxidative stress, namely, by modulating the expression of UCP2.

## Methods

### Animal treatment

Matched TRPV1 knockout (TRPV1^-/-^) and C57BL/6J wild type (WT) mice were purchased from Jackson Laboratory (Bar Harbor, ME, USA), as well as matched db/db and C57BL/KsJ WT mice. Matched UCP2 knockout (UCP2^-/-^) and C57BL/6J WT mice were provided by Chenyu Zhang (Model Animal Research Center, Nanjing University, Nanjing, China). All mice were housed under a 12 h/12 h day/night cycle with free access to food and water. In addition, db/db and WT mice were fed with normal chow plus 0.01% capsaicin for 14 weeks. The numbers of mice are 6 in each group. Blood pressure of db/db mice measured by the tail-cuff method consciously after 14 weeks period dietary administration. The Institute’s Animal Care and Use Committee approved all animal protocols.

### Preparation of aortic and mesenteric arteries

After animals were sacrificed, the thoracic aortas and mesenteric arteries were dissected as previous reported [20], carefully freed from connective tissue, and placed in Krebs solution, containing (in mmol/L) NaCl 119; NaHCO_3_ 25; glucose 11.1; KCl 4.7; KH_2_PO_4_ 1.2; MgSO_4_ 1.2; CaCl_2_ 2.5; pH 7.4.

### Measurement of vascular activities

Changes in the isometric tone of the aortic and mesenteric artery rings were recorded by four-chamber wire myograph (model 610M; Danish Myo Technology, Aarhus, Denmark), as previously described [11,22]. The arterial segments were stretched to an optimal baseline tension and then allowed to equilibrate for one hour before being contracted with 60 mmol/L KCl and rinsed in Krebs solution. Endothelium-dependent relaxation was measured by testing the concentration-response relationship upon the cumulative addition of acetylcholine (Ach, 1 nmol/L to 10 μmol/L) to phenylephrine (Phe, 1 nmol/L to 10 μmol/L)-precontracted rings. In some cases, arteries were incubated with the eNOS inhibitor NG-nitro-L-arginine methyl ester (L-NAME, 100 μmol/L, 30 minutes) before Phe-stimulated precontraction. The endothelium-independent relaxation response to nitroglycerine (NTG) was also measured in artery rings.

### Artery and endothelial cell culture

Mouse aortic rings were dissected in sterile PBS and incubated in DMEM supplemented with 10% foetal bovine serum (FBS, Gibco), 100 IU/mL penicillin and 100 μg/mL streptomycin. The high-glucose (HG: 30 mmol/L) condition was achieved by the addition of 24.5 mmol/L glucose, while 24.5 mmol/L of mannitol was used in the normal-glucose (NG) osmotic control condition [1]. After the incubation period, the aortic rings were transferred to a chamber filled with fresh Krebs solution and mounted in a myograph to measure changes in the isometric force [23]. Porcine iliac artery endothelial cells (PIECs) were obtained from the Institute of Biochemistry and Cell Biology [24]. The cells were grown in DMEM supplemented with 10% FBS and 1% antibiotics. Cultured arteries and cells were maintained at 37°C in a humidified atmosphere of 95% O_2_/5% CO_2_. The cells were made quiescent by incubation of 90% confluent cell cultures in serum-free DMEM and were incubated with capsaicin (1 *μ*mol/L) for 12 h in the presence or absence of TRPV1 antagonist 5’-iodo-resiniferatoxin (iRTX, 1 *μ*mol/L), PKA inhibitor KT5720 (2 *μ*mol/L) and UCP2 inhibitor genipin (10 *μ*mol/L), concentrations of these chemicals based on our previous reports [18,20].

### Dihydroethidium assay

To assess superoxide production, dihydroethidium (DHE) staining was performed according to a previously described method [25]. Mesenteric arteries were isolated and embedded in tissue-freezing compound. The specimens were cut into 10 μm sections and transferred to cover glass before being incubated with DHE diluted in Krebs solution for 45 min at 37°C, after which the sections were washed three times in DHE-free Krebs solution. To quantify DHE fluorescence, the glass slides were placed under an inverted fluorescence microscope (Nikon TE2000; Nikon Corporation, Tokyo, Japan) outfitted with a×40 Plan Fluor objective. Images were acquired using NIS-Elements 3.0 software (Nikon), and the fluorescence intensity was analysed.

### Evaluation of NO levels

The NO levels in vessels and PIECs were assessed by staining with DAF-2 DA in Krebs solution for 45 min at 37°C followed by three washes with Krebs solution. Mesenteric arteries were prepared as described above. The NO fluorescence was detected and the fluorescence intensity was analysed as described above.

### Immunofluorescence staining

Aortic segment were fixed with 10% formalin at room temperature for 60 min and then bathed in a 2% hydrogen peroxide methanol solution for 30 min. The vessels were incubated with antibodies against TRPV1 (Alomone Labs, Israel), PKA or UCP2 (Santa Cruz Biotechnology, USA) overnight at 4°C and incubated with fluorescent dye-labeled secondary antibodies (ZSGB-BIO, China) at room temperature for 30 min. Images were obtained with a TE2000-U Nikon eclipse microscope and analyzed with NIS-Elements imaging software [13].

### Western blot analysis

Immunoblots of TRPV1, PKA, p-PKA, AMPK, UCP2, p22^phox^, p-eNOS and GAPDH were prepared as previously described [20]. After incubation with secondary antibodies (ZSGB-BIO, China) at room temperature for 2 h, the proteins were detected with enhanced chemiluminescence and quantified using a Gel Doc 2000 Imager (Bio-Rad, USA). Protein expression was normalized to the internal control, GAPDH. All of the primary antibodies were purchased from Santa Cruz Biotechnology (Santa Cruz, CA, USA).

### Drugs

Ach, Phe, L-NAME, NTG, capsaicin, iRTX, KT5720, genipin, DHE and DAF-2 DA were purchased from Sigma-Aldrich, St. Louis, MO, USA.

### Statistical analysis

Data are mean ± SEM. The maximum response (*E*max) were calculated from individual agonist concentration–response curves using GraphPad Prism 3.0 (GraphPad Software, San Diego, CA). The statistical differences in mean values were assessed by the Student’s *t*-test. Two-sided *P* values < 0.05 were considered as statistically significant.

## Results

### Activation of TRPV1 upregulates UCP2 through PKA phosphorylation

**Figure 1:**
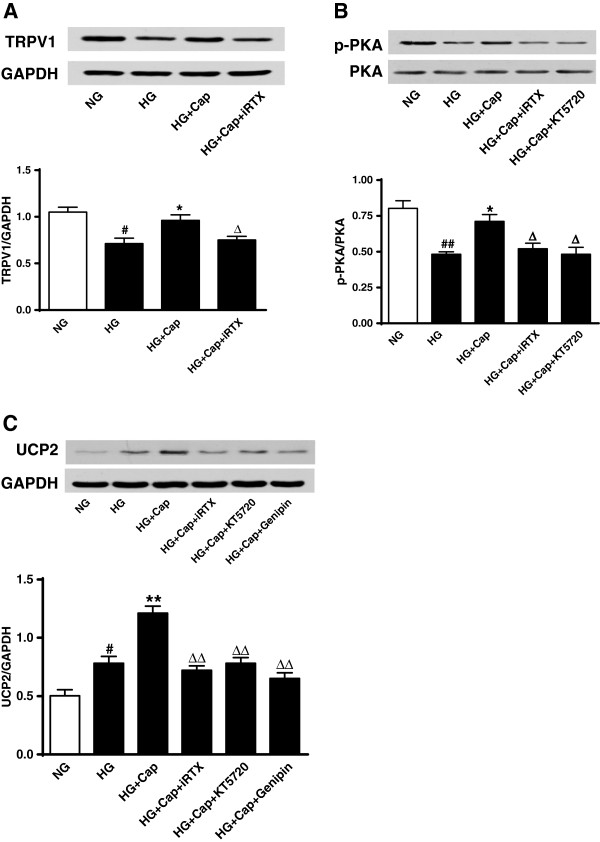
**Activation of TRPV1 up-regulates UCP2 through PKA phosphorylation. A**, **B** and **C**: Representative western blot images showing protein expressions of TRPV1 (**A**), p-PKA/PKA (**B**) and UCP2 (**C**) in endothelial cells cultured with normal-glucose (NG, glucose 5.5 mmol/L), high-glucose (HG, glucose 30 mmol/L), HG+capsaicin (HG+Cap, Cap 1 μmol/L), HG+Cap+5’-iodo-resiniferatoxin (HG+Cap+iRTX, iRTX 1 μmol/L), HG+Cap+KT5720 (2 μmol/L) or HG+Cap+Genipin (10 μmol/L); *#P<0.05, ##P<0.01* versus NG group; *P<0.05, **P<0.01 versus HG group; Δ*P<0.05,* ΔΔ*P<0.01* versus HG+Cap group. Data are mean ± SEM. Each n = 3.

We first showed that exposure to a high level of glucose (30 mmol/L) decreased TRPV1 expression and PKA phosphorylation in cultured endothelial cells compared with control cells and that these effects can be reversed by the administration of capsaicin (1 μmol/L). In contrast, the effects of capsaicin were blocked by the addition of the specific TRPV1 antagonist iRTX (1 μmol/L) and the PKA inhibitor KT5270 (2 μmol/L) (Figure 1A and B). In addition, high-glucose conditions increased the endothelial UCP2 level. Administration of capsaicin further increased the level of UCP2 in the cultured endothelial cells incubated in high-glucose conditions, but again, this effect of capsaicin was inhibited by antagonists of TRPV1 and inhibitor of PKA and by the UCP2 inhibitor, genipin (10 μmol/L) (Figure 1C). Genipin significantly reduced UCP2, but iRTX and KT5720 did not affect UCP2 levels in normal-glucose solution (Additional file 1: Figure S1). These results indicated that the upregulation of UCP2 occurred through a TRPV1 activation-mediated PKA pathway.

### Effect of TRPV1 activation on the production of ROS and NO through the PKA/UCP2 pathway

**Figure 2:**
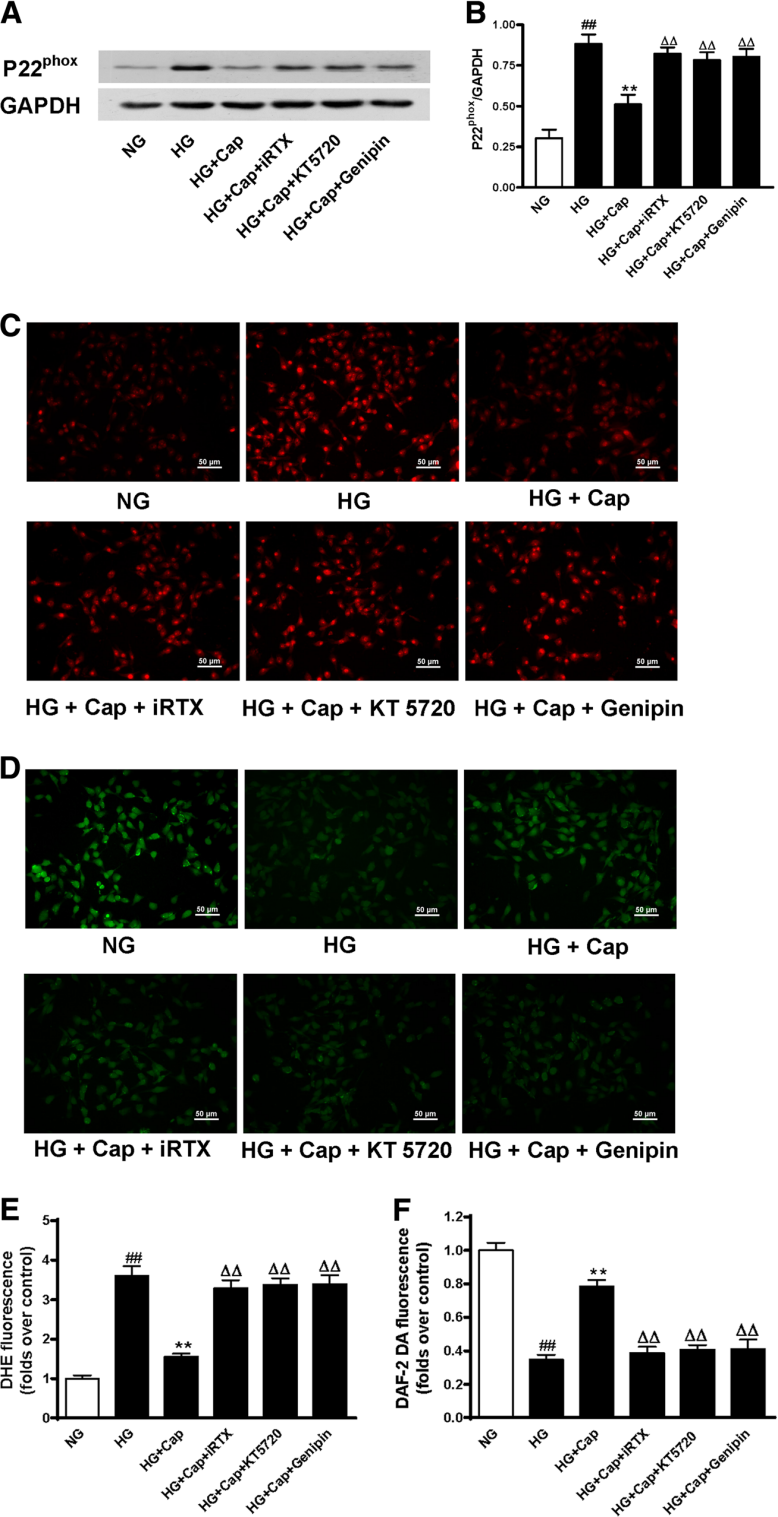
**The effect of TRPV1 activation on the production of ROS and NO through the PKA/UCP2 pathway. A** and **B**: Representative western blot images (**A**) and summary data (**B**) showing P22^phox^ protein level in endothelial cells cultured with normal-glucose (NG, glucose 5.5 mmol/L), high-glucose (HG, glucose 30 mmol/L), HG+capsaicin (HG+Cap, Cap 1 μmol/L), HG+Cap+5’-iodo-resiniferatoxin (HG+Cap+iRTX, iRTX 1 μmol/L), HG+Cap+KT5720 (2 μmol/L), HG+Cap+Genipin (10 μmol/L). ##*P <0.01* versus NG group; **P <0.01 versus HG group; ΔΔ*P <0.01* versus HG+Cap group; Data are mean ± SEM. Each n = 3. **C**-**F**: Representative endothelial cells stained by DHE (**C** and **E**) and DAF-2 DA (**D** and **F**) cultured with NG, HG, HG+Cap, HG+Cap+iRTX, HG+Cap+KT5720, HG+Cap+Genipin. *##P <0.01* versus NG group; **P <0.01 versus HG group; ΔΔ*P <0.01* versus HG+Cap group. Data are mean *±* SEM from 4 independent experiments. The scale bar indicates 50 μm.

NAD(P)H oxidase has been implicated as the major source of ROS generation and plays a role in eNOS uncoupling in the vasculature in response to high-glucose conditions [26]. UCP2 is a physiological regulator of mitochondrial ROS generation. We showed that high-glucose exposure significantly increased the p22^phox^ subunit of NAD(P)H oxidase, but this effect was blunted by capsaicin treatment in the cultured endothelial cells. Furthermore, the inhibition of TRPV1, PKA and UCP2 using their respective antagonists abolished the effects of capsaicin (Figure 2A and B). Also, these effects were observed in endothelial cells on normal-glucose condition (Additional file 1: Figure S2A). In addition, high-glucose conditions increased the production of ROS, which was assessed using dihydroethidium (DHE) fluorescence, and reduced the level of NO, which was evaluated by DAF-2 DA fluorescence. However, capsaicin treatment decreased the production of ROS and increased NO levels in the cultured endothelial cells that were exposed to high-glucose conditions, and these effects were antagonised by the inhibition of TRPV1, PKA and UCP2 (Figure 2C-F). Moreover, iRTX, KT5720 or genipin increased ROS level and decreased NO production of endothelial cells on high-glucose condition separately (Additional file 1: Figure S2B). These results suggest that TRPV1 activation by capsaicin reduces the level of ROS and increases NO production via the PKA/UCP2 pathway.

### TRPV1 activation ameliorates high-glucose-induced endothelial dysfunction in a UCP2-dependent manner

**Figure 3:**
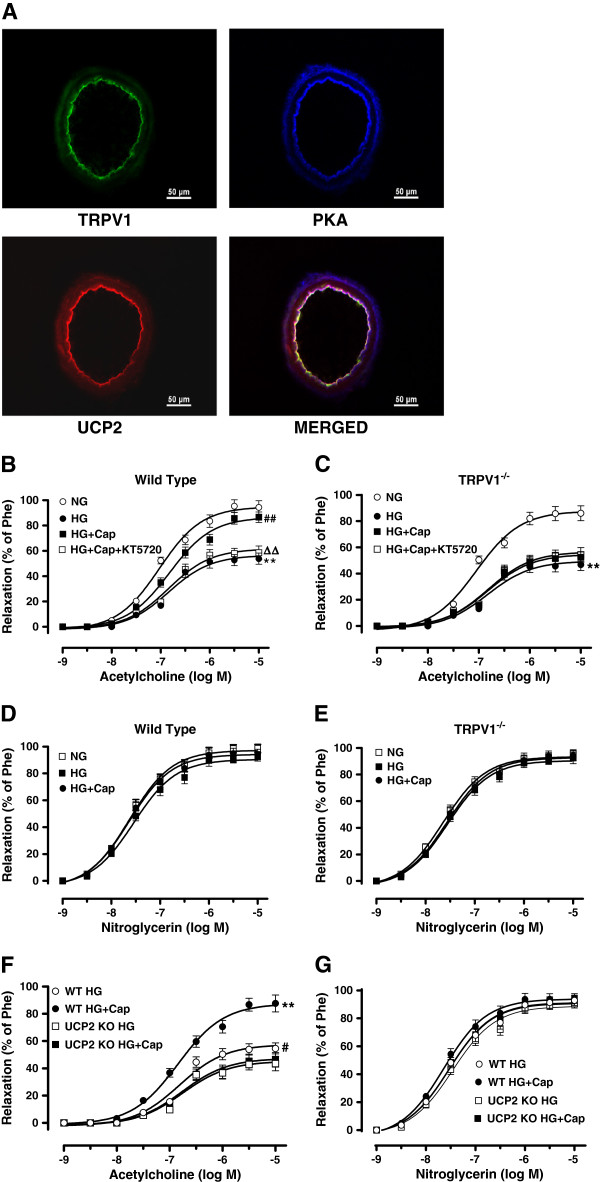
**TRPV1 activation ameliorates high-glucose-induced endothelial dysfunction in a UCP2-dependent manner. A**: Representative immunofluorescence images showing the co-expression of TRPV1, PKA and UCP2 in the aortas from wild type mice, particularly in the endothelium (Bar denotes 50 μm). **B** and **C**: Acetylcholine (1 nmol/L to 10 μmol/L)-induced endothelium-dependent relaxation of isolated aortic artery rings from wild type and TRPV1^-/-^ mice, pre-incubated with normal-glucose for 12 hours (NG, glucose 5.5 mmol/L), high-glucose (HG, glucose 30 mmol/L), HG+capsaicin (HG+Cap, Cap 1 μmol/L), HG+Cap+KT5720 (2 μmol/L); **P<0.01 versus NG group; #*#P<0.01* versus HG group; ΔΔ*P<0.01* versus HG+Cap group. Data are mean ± SEM. Each n=6. **D** and **E**: Nitroglycerin (1 nmol/L to 10 μmol/L) -induced endothelium-independent relaxation of isolated aortic artery rings from wild type and TRPV1^-/-^ mice, after cultured for 12 hours with NG, HG, HG+Cap. Data are mean ± SEM. Each n =6. **F** and **G**: Representative data that Acetylcholine- and nitroglycerin-induced relaxation in the presence or absence of capsaicin (Cap, 1 μmol/L) in isolated aortic arteries rings from UCP2^-/-^ mice and wild type (WT) mice under high-glucose condition(HG). **P<0.01 HG + Cap versus HG group of WT, *#P<0.05* HG group of WT versus HG group of UCP2^-/-^. Data are mean ± SEM. Each n=6.

To further determine the role of UCP2, we investigated the vasodilatation effect of capsaicin on aortic artery rings *ex vivo* under high-glucose conditions. Immunofluorescence images clearly showed the distribution of TRPV1, PKA and UCP2 in the vascular endothelium (Figure 3A). High-glucose (HG) exposure for 12 h impaired the endothelium-dependent relaxation of aortic rings from both WT and TRPV1^-/-^ mice as compared with the normal-glucose (NG) exposure condition. Under HG conditions, incubation in the presence of capsaicin for 12 h improved the endothelium-dependent relaxation of aortic rings from WT mice but not that of aortic rings from TRPV1^-/-^ mice (Figure 3B and C). We next used the UCP2^-/-^ mice and their matched WT counterparts to explore the role of UCP2 in TRPV1 activation-mediated effects. Incubation with capsaicin did not improve the endothelium-dependent relaxation of aortas from UCP2^-/-^ mice under high-glucose conditions (Figure 3F). The endothelium-independent relaxations did not differ among the groups tested (Figure 3D, E and G). Exposure to high-glucose condition decreased TRPV1 expression and phosphorylation of PKA, but increased the UCP2 level in cultured aortas compared with control aortas in normal-glucose, these effects can be reversed by capsaicin administration. However, capsaicin administration further elevated the level of UCP2 in the cultured endothelial aortas under high-glucose condition (Additional file 1: Figure S3A). Acetylcholine induced endothelial-dependent vasodilation of UCP2^-/-^ mice was weakened by pre-cultured with high-glucose for 12h (Additional file 1: Figure S3B). These results indicate that TRPV1 activation ameliorates high-glucose-induced endothelial dysfunction in a UCP2-dependent manner.

### TRPV1 activation by dietary capsaicin promotes endothelial PKA phosphorylation and increases UCP2 levels in diabetic mice

**Figure 4:**
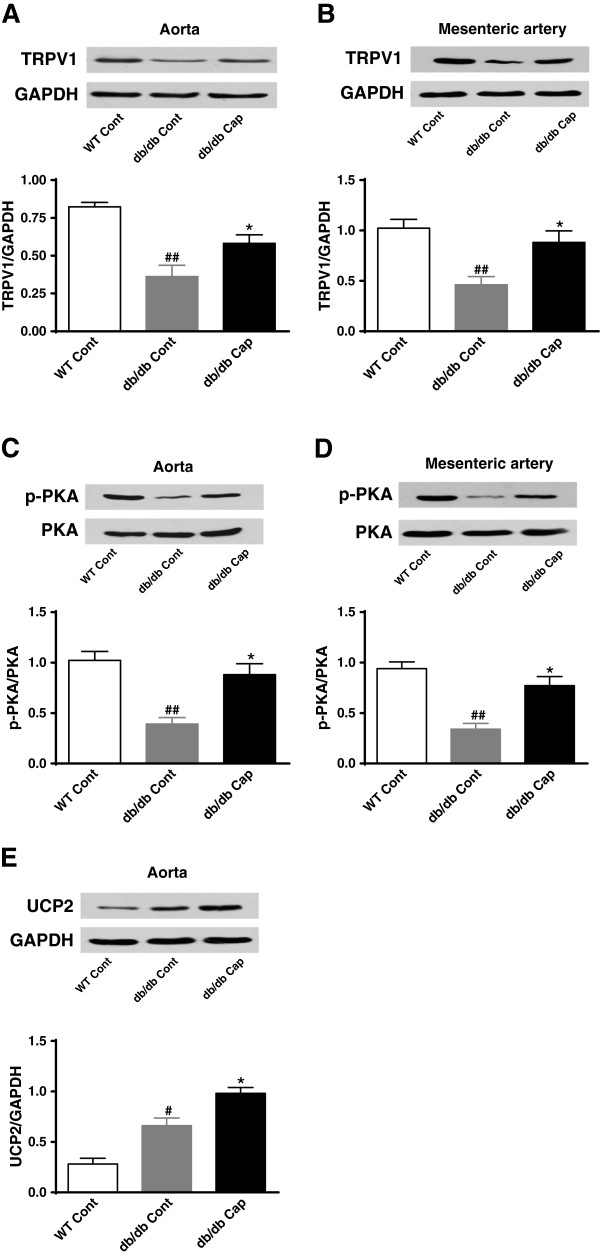
**TRPV1 activation by dietary capsaicin promotes endothelial PKA phosphorylation and increases UCP2 levels in diabetic mice.** Representative protein expression of TRPV1 (**A** and **B**), p-PKA/PKA (**C** and **D**) and UCP2 (**E**) levels in aorta or mesenteric arteries from db/db mice treated with normal diet (db/db Cont) or normal diet plus 0.01% capsaicin (db/db Cap) and the lean littermate control C57BL/KsJ mice treated with normal diet (WT Cont). Data are mean ± SEM. Each n = 3. ##*P <0.01,* #*P <0.05* versus WT Cont group; *P <0.05 versus db/db Cont group.

We investigated whether dietary capsaicin has a beneficial effect on UCP2 level in the vasculature. Diabetic obese mice (db/db) and wild-type lean littermate control mice (C57BL/KsJ) were examined. TRPV1 and phosphorylation of PKA, but not total PKA were significantly down-regulation in the aorta and mesenteric arteries from db/db mice compared to lean wild type mice, but increased after dietary capsaicin administration for 14 weeks (Figure 4A-D). We found that UCP2 level was significantly higher and administration of dietary capsaicin was further elevated in the aorta from db/db mice compared to lean wild type mice (Figure 4E). These results indicate that TRPV1 activation by dietary capsaicin promotes endothelial PKA phosphorylation and increases the expression level of UCP2 in diabetic mice.

### TRPV1 activation by dietary capsaicin attenuates endothelial oxidative stress and increases NO levels in diabetic mice

**Figure 5:**
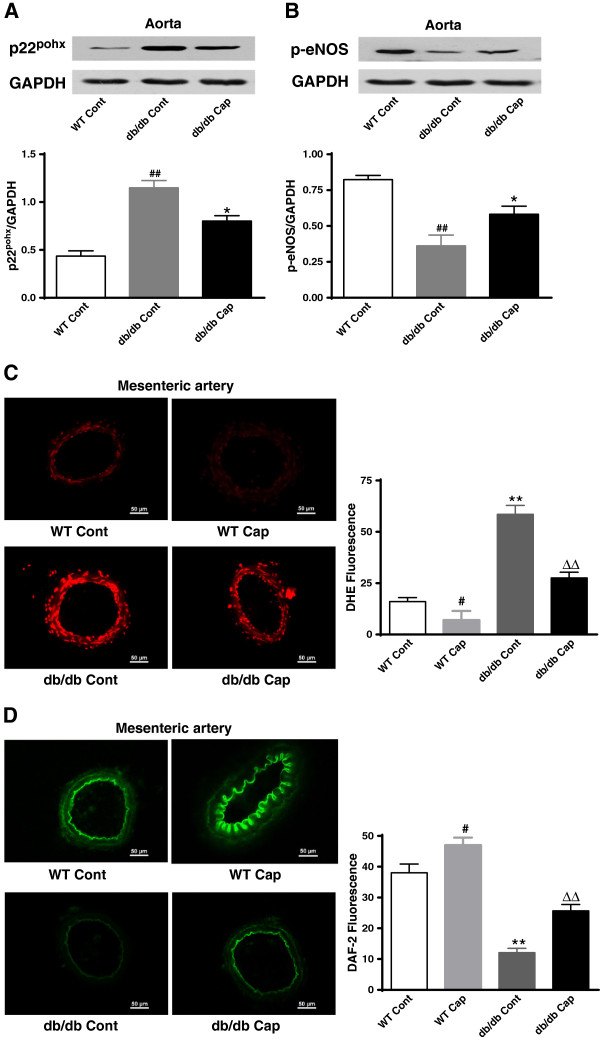
**TRPV1 activation by dietary capsaicin attenuates endothelial oxidative stress and increases the level of NO in diabetic mice. A** and **B**: Representative protein expressions of p22^phox^ (**A**) and p-eNOS (**B**) in aortas from db/db mice treated with normal diet (db/db Cont) or normal diet plus 0.01% capsaicin (db/db Cap) and wild type mice treated with normal diet (WT Cont). ##*P<0.01* versus the WT Cont group; *P<0.05 versus the db/db Cont group. Data are mean ± SEM. Each n = 3. **C** and **D**: Representative images and summary data detected by DHE (**C**) and DAF-2 DA (**D**) in mesenteric arteries from wild type and db/db mice treated with normal diet (WT Cont and db/db Cont) or normal diet plus 0.01% capsaicin (WT Cap and db/db Cap). #P<0.05 versus WT Cont group; ** P<0.01 versus WT Cap group; ΔΔP<0.01 versus db/db cont group. Data are mean ± SEM. Each n = 4. The scale bar indicates 50 μm.

We next investigated the effects of PKA phosphorylation and UCP2 upregulation on oxidative stress and endothelial NO production in mesenteric arteries. Immunoblotting showed that dietary capsaicin decreased the vascular expression of p22^phox^ and increased the level of p-eNOS in the aorta in db/db mice (Figure 5A and B). Additionally, chronic dietary capsaicin significantly reduced superoxide anion production and increased NO production in the mesenteric arteries in db/db mice and wild type mice (Figure 5C-D). These results indicate that long-term administration of dietary capsaicin ameliorates vascular oxidative stress and increases NO levels in diabetic mice.

### Dietary capsaicin improves endothelium-dependent relaxation in db/db mice

**Figure 6:**
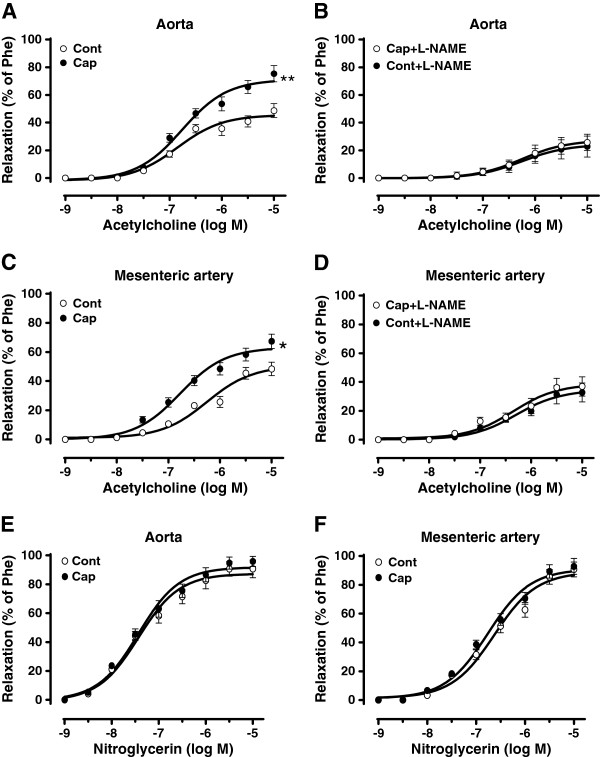
**Dietary capsaicin improves the endothelium-dependent relaxation in db/db mice. A** and **C**: Quantification of the endothelium-dependent relaxation induced by acetylcholine treatment (1 nmol/L to 10 μmol/L) of aortas and mesenteric arteries from db/db mice given either a normal diet (Cont) or a normal diet plus 0.01% capsaicin (Cap) for 14 weeks. Data are expressed as the mean ± SEM from six rings, with six mice per group. *P<0.05, ** P<0.01 versus the db/db control group. **B** and **D**: The inhibitory effect of nitro-L-arginine methyl ester (L-NAME, 100 μM, 30min), on acetylcholine-induced maximal relaxation in aortas and mesenteric arteries from db/db mice with or without dietary capsaicin administration. Data are expressed as the mean ± SEM from six rings, with six mice per group. **E** and **F**: Representative data showing endothelium-independent relaxation in db/db mouse aortas and mesenteric arteries with or without dietary capsaicin administration. Data are expressed as the mean ± SEM from six rings, with six mice per group.

Dietary capsaicin did not affect the blood pressure (data not shown) but markedly augmented the endothelium-dependent relaxation observed in both the aorta and mesenteric arteries (MA) of db/db mice (Aorta Emax(%): 45.59 ± 2.35 in control vs 71.07 ± 3.02 in capsaicin-administered mice, *P<0.01*; MA Emax(%): 50.36 ± 3.73 in control vs 62.94 ± 2.68 in capsaicin-administered mice, *P<0.05*) (Figure 6A and C). This difference was abolished by pretreatment with L-NAME (Aorta Emax(%): 24.02 ± 2.43 in control vs27.25 ± 2.36 in capsaicin-administered mice, *P>0.05*; MA Emax(%): 37.56 ± 3.41 in control vs 45.01 ± 5.61 in capsaicin-administered mice, *P>0.05*) (Figure 6B and D). Endothelium-independent relaxation induced by nitroglycerine did not differ among the groups tested (Figure 6E and F). These findings indicate that prolonged administration of dietary capsaicin promotes endothelium-dependent relaxation in diabetic mice.

## Discussion

The major findings from this study are described here. First, upregulation of UCP2 by capsaicin reduced the production of ROS and increased the levels of NO in cultured endothelial cells through a TRPV1 activation-mediated PKA pathway. Second, TRPV1 activation by capsaicin ameliorated high-glucose-induced endothelial dysfunction in a UCP2-dependent manner. Third, prolonged administration of dietary capsaicin increased the vascular levels of PKA phosphorylation and of UCP2 expression, ameliorated vascular oxidative stress and improved endothelium-dependent relaxation in diabetic mice.

TRPV1 plays a role in promoting insulin secretion in beta cells [27,28] and the stimulation of insulin receptors in the vasculature affects endothelial function [29-31]. Our previous study showed that TRPV1 activation-stimulated GLP-1 secretion could be a promising approach for the intervention of diabetes [19]. Furthermore, current study also shows that activation TRPV1 by capsaicin can increase UCP2 expression [13,14]. Oktavianthi, et al demonstrated the importance of common UCP2 gene polymorphisms in the development of obesity in a Balinese population [32]. Treatment with 6 mg/d capsinoids orally was associated with abdominal fat loss [33]. Capsaicin is a specific agonist of TRPV1 channels and dose dependently induced calcium influx in endothelial cells and in freshly isolated mesenteric arteries [17,20]. Several studies show that hyperglycemia cause a dysfunction and down-regulation of TRPV1 in diabetic animals [34-36]. In addition, high-glucose reduced phosphorylation of PKA [37] and increased UCP2 level in pancreatic islets of animal models of type 2 diabetes [38]. Our results from db/db mice are agreement with these studies.

The underlying mechanisms of cardiovascular complications in diabetic patients are incompletely understood [2]. However, hyperglycemia plays a pivotal role in endothelial cell dysfunction, and induce mitochondrial ROS generation contribute to diabetic vascular lesions [2,4]. The increased activation of proinflammatory factors is related to hyperglycemic damage, which is mainly caused by a hyperglycemia-induced overproduction of superoxide anion through mitochondrial electron transport chain [39]. Dhamrait.S, et al, showed that UCP2 expression is induced by oxidative stress, which protecting against further ROS generation. Increased UCP2 activity may therefore limit ROS generation, decreasing atherosclerotic risk in diabetic man [40]. Thus, an elevated UCP2 in response to an elevation in superoxide under high-glucose condition plays an active role in feedback regulation of ROS production associated with oxidative stress [41] Antagonising ROS by treatment with antioxidants such as tempol promotes endothelium-dependent vasodilatation in hypertensive and diabetic animal models [42,43]. Several studies have shown that UCP2 deficiency causes vascular dysfunction and target organ damage in stroke, atherosclerosis and hypertension [7,12,42]. NADPH oxidases are major sources of ROS in the vasculature. NADPH oxidase consists of six subunits: two trans-membrane units, p22^phox^ and gp91^phox^, and four cytosolic units, p47^phox^, p67^phox^, p40^phox^ and the small GTPase rac1 or rac2.High-glucose levels stimulate ROS production through the activation of NADPH oxidase [2,4,44]. The expression of p22^phox^ is significantly increased in rat and human diabetic arteries [4]. Over-expression of UCP2 can protect the vasculature against the effects of high-glucose exposure by inhibiting NADPH oxidase activity [8].

The present study demonstrates that the administration of capsaicin can significantly increase the vascular expression level of UCP2, both in the presence of high-glucose levels and in diabetic mice. Furthermore, high-glucose- or hyperglycemia-induced ROS production and endothelial dysfunction can be blunted by the administration of capsaicin. We further investigated the mechanism responsible for the protective role of capsaicin observed in this study. It has been shown that several protein kinases, such as adenosine monophosphate activated protein kinase (AMPK) and PKA, modulate UCP2 expression and decrease ROS production [8,27,45]. Furthermore, adiponectin reverses hyperglycemia-associated endothelial ROS generation via a cAMP/PKA pathway [16]. Our previous studies showed that dietary capsaicin improved endothelium-dependent vasorelaxation in a hypertensive rat genetic model and ameliorated atherosclerosis in apolipoprotein E deficient (ApoE^-/-^) mice through the promotion of PKA phosphorylation [18]. The present study examined the co-expression of TRPV1, PKA and UCP2 in the vasculature. Administration of capsaicin resulted in a remarkable up-regulation of UCP2 expression through a TRPV1-mediated PKA pathway. Furthermore, the high-glucose-induced impairment of vasorelaxation observed in aortic rings from UCP2 knockout mice was not ameliorated by capsaicin administration. Our study indicates that capsaicin treatment prevents high-glucose-induced endothelial cell dysfunction through TRPV1/PKA pathway-mediated upregulation of UCP2, which ultimately leads to a reduction in oxidative stress and protects endothelial cells from damage.

### Limitations

There are several limitations in this study. Long-term consumption of capsaicin may affect brain or stomach. The effects of capsaicin on these organs need to be clarified in future. It is worthy to further validate the benefit effect of dietary capsaicin on endothelial function in diabetic patients.

## Conclusion

**Figure 7:**
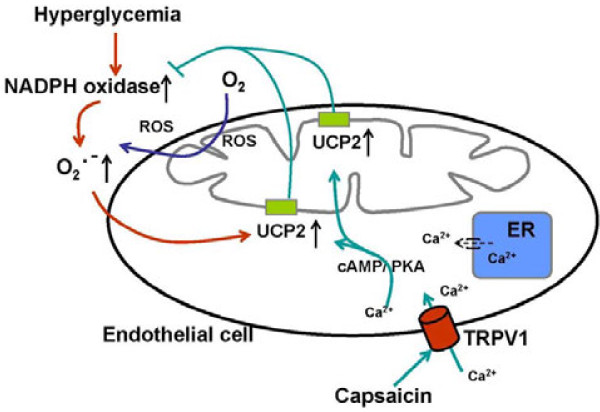
**Upregulation of UCP2 by TRPV1 activation attenuates hyperglycemia-induced ROS production in ECs.** The increased metabolism of glucose due to intracellular hyperglycemia leads to the overproduction of NADH, a critical component of the superoxide-generating mechanism in endothelial cells. Upregulation of mitochondrial UCP2 in response to elevated superoxide levels plays an active role in the feedback regulation of reactive oxygen species production that is associated with chronic oxidative stress. Activation of the endothelial TRPV1 channel in endothelial cells by dietary capsaicin mediates the phosphorylation of PKA and upregulates UCP2, thus inhibiting the activity of NADPH and decreasing ROS production in ECs.

In summary, this study shows that the administration of capsaicin can reverse high-glucose-induced endothelial dysfunction through TRPV1 activation. Our mechanistic evidence suggests that this vascular benefit is likely to result from an enhancement of the phosphorylation of PKA and from the upregulation of UCP2 expression, which thus reduces oxidative stress and increases the level of NO. This effect accounts for the endothelium-dependent relaxation observed in capsaicin-treated db/db mice (Figure 7). Our findings provide new insight into activation of TRPV1 by dietary capsaicin may represent a promising lifestyle intervention and beneficial to the vascular complications in diabetic patients.

## Abbreviations

UCP2: Uncoupling protein 2; ROS: Reactive oxygen species; TRPV1: Transient receptor potential vanilloid 1; WT: Wild type; PKA: Protein kinase A; NO: Nitric oxide; DHE: Dihydroethidium; NAD(P)H: Nicotinamide adenine dinucleotide phosphate; eNOS: Endothelial nitric oxide synthase; cAMP: Cyclic adenosine monophosphate; NTG: Nitroglycerine; NG: Normal-glucose; HG: High-glucose; L-NAME: NG-nitro-L-arginine methyl ester; PIECs: Porcine iliac artery endothelial cells; iRTX: 5’-iodo-resiniferatoxin: M, mol/L.

## Competing interests

The authors declare that they have no competing interests.

## Authors’ contributions

JS and YFP performed most of the experiments and analyzed data and wrote the manuscript. PJW performed some experiments and edited the manuscript. SJC reviewed and edited the manuscript. YZ and CL performed some experiments and contributed to the discussion. ZMZ and QHS edited the manuscript and contributed to the discussion. DYL designed the experiments and wrote and edited the manuscript. The authors thank Lijuan Wang (Chongqing Institute of Hypertension, China) for technical assistance. All authors read and approved the final manuscript.

## Supplementary Material

Additional file 1: Figure S1 Effect of iRTX, KT5720 and genipin on UCP2 of endothelial cells on normal-glucose condition. **Figure S2**: A. Effect of capsaicin on p22^phox^ of endothelial cells on normal-glucose condition. B. Effect of iRTX, KT5720 and genipin on ROS level and NO production of endothelial cells on high-glucose condition. **Figure S3**: A. Effect of capsaicin on TRPV1, p-PKA/PKA and UCP2 in aortas of rats on high-glucose condition. B. Endothelial-dependent vasodilation of UCP2 KO mice in normal or high-glucose solution. Click here for file
