# Supplementary material for: TRPV1-mediated UCP2 upregulation ameliorates hyperglycemia-induced endothelial dysfunction
Source: Cardiovasc Diabetol. 2013 Apr 22;12:69. doi: 10.1186/1475-2840-12-69 (PMC3644255; doi:10.1186/1475-2840-12-69)

Supplementary Figure 1

Effect of iRTX, KT5720 and genipin on UCP2 of endothelial cells on normal-glucose condition.

Representative western blot images showing UCP2 protein expression in endothelial cells cultured with normal-glucose (NG, glucose 5.5 mmol/L), NG+5'-iodo-resiniferatoxin (iRTX, antagonist of TRPV1, 1 μmol/L) (NG+ iRTX), NG+ KT5720 (PKA inhibitor, 2 μmol/L), NG+Genipin (UCP2 inhibitor, 10 μmol/L). *P<0.05 versus NG group. Data are mean ± SEM for 3 experiments.

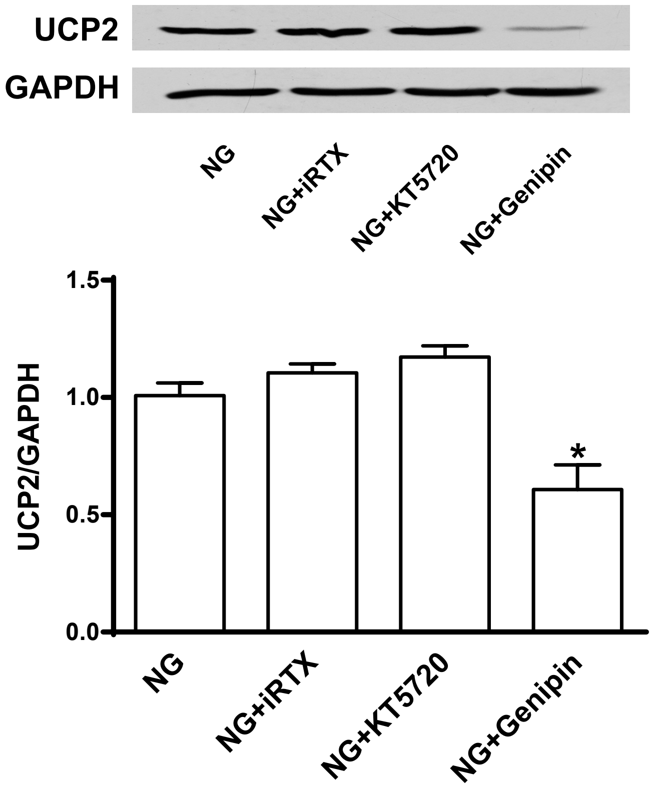

Supplementary Figure 2

A. Effect of capsaicin on p22^{phox} of endothelial cells on normal-glucose condition.

Representative western blot images showing p22^{phox} protein expression in endothelial cells cultured with normal-glucose (NG, glucose 5.5 mmol/L), NG+capsaicin (Cap, 1 μmol/L) (NG+Cap), NG+5'-iodo-resiniferatoxin (iRTX, antagonist of TRPV1, 1 μmol/L) (NG+ iRTX), NG+ KT5720 (PKA inhibitor, 2 μmol/L), NG+Genipin (UCP2 inhibitor, 10 μmol/L). *P<0.05 versus NG group. Data are mean ± SEM for 3 experiments.

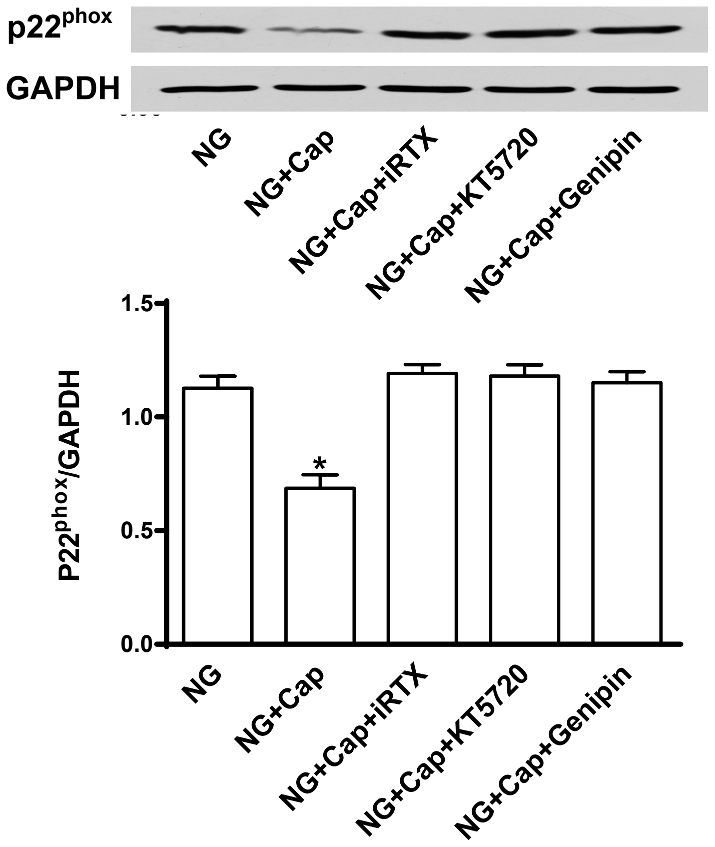

Supplementary Figure 2

B. Effect of iRTX, KT5720 and genipin on ROS level and NO production of endothelial cells on high-glucose condition.

Representative images showing ROS level detected by DHE (red) and NO production detected by DAF 2-DA (green) in endothelial cells cultured with high-glucose (HG, glucose 30 mmol/L), HG+5'-iodo-resiniferatoxin (iRTX, antagonist of TRPV1, 1 μmol/L) (HG+ iRTX), HG+ KT5720 (PKA inhibitor, 2 μmol/L), HG+Genipin (UCP2 inhibitor, 10 μmol/L). *P<0.05 versus HG group. Data are mean ± SEM for 6 experiments.

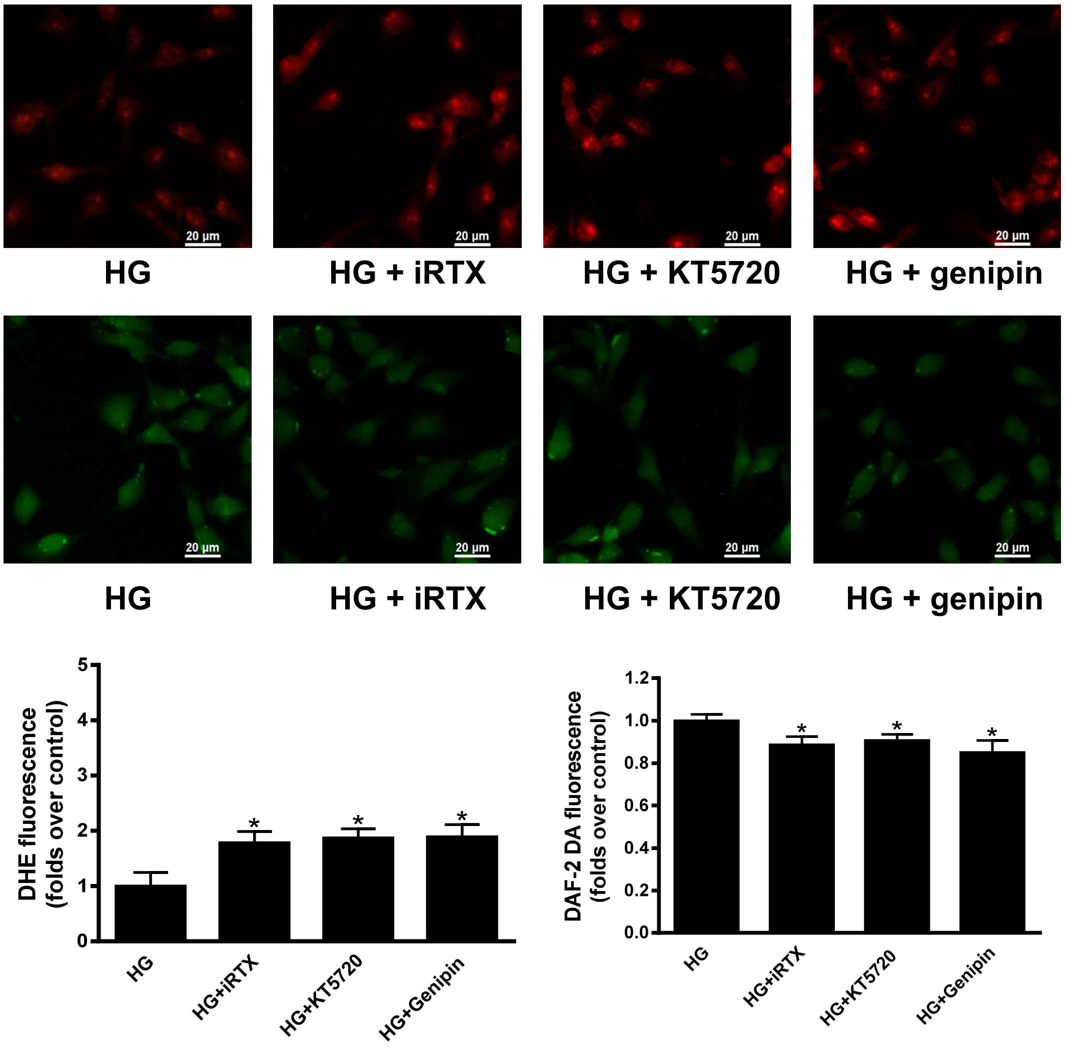

Supplementary Figure 3

A. Effect of capsaicin on TRPV1, p-PKA/PKA and UCP2 in aortas of rats on high-glucose condition.

Representative western blot images showing TRPV1, p-PKA/PKA and UCP2 protein expression in aortas of SD rats. NG: aortas cultured with normal-glucose (NG, glucose 5.5 mmol/L) for 12 h; HG: aortas cultured with high-glucose (HG, 30 μmol/L) for 12 h; HG+Cap: first aortas incubated 12 h in HG then another 12 h in HG+capsaicin (Cap, 1 μmol/L) .*P<0.05 versus NG group, #P<0.05 versus HG group. Data are mean ± SEM for 3 experiments.

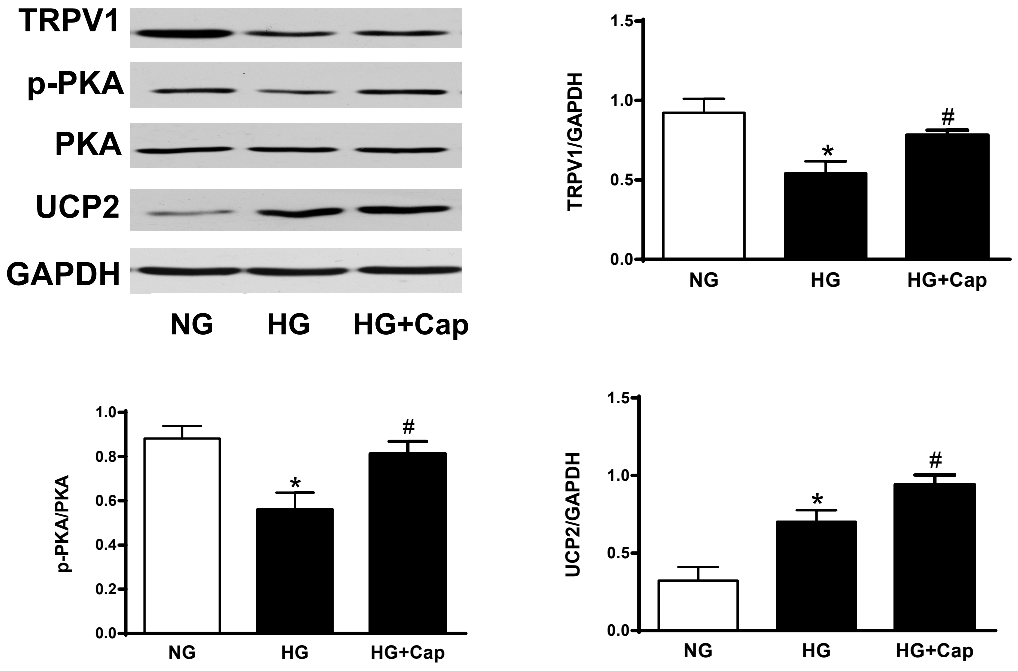

Supplementary Figure 3

B. Endothelial-dependent vasodilation of UCP2 KO mice in normal or high-glucose solution

Acetylcholine induced Endothelial-dependent mesenteric arteries relaxtion of UCP2 KO mice in normal-glucose (NG, glucose 5.5 mmol/L) or high-glucose (HG, glucose 30 mmol/L) solution. **P<0.01 versus HG group. Data are mean ± SEM. Each n=6.

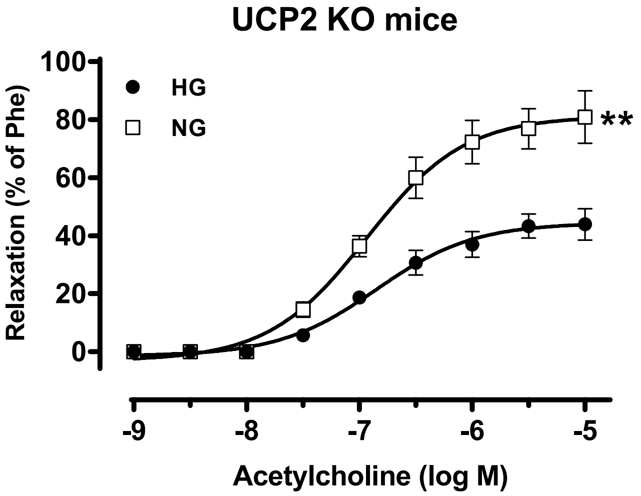

Supplement: Additional file 1: Figure S1 — Effect of iRTX, KT5720 and genipin on UCP2 of endothelial cells on normal-glucose condition. Figure S2: A. Effect of capsaicin on p22phox of endothelial cells on normal-glucose condition. B. Effect of iRTX, KT5720 and genipin on ROS level and NO production of endothelial cells on high-glucose condition. Figure S3: A. Effect of capsaicin on TRPV1, p-PKA/PKA and UCP2 in aortas of rats on high-glucose condition. B. Endothelial-dependent vasodilation of UCP2 KO mice in normal or high-glucose solution. [file 1475-2840-12-69-S1.pdf]
